# Opposing roles of ICAT and Wnt/β-catenin signaling in NSC67657-induced monocytic differentiation

**DOI:** 10.18632/oncotarget.19457

**Published:** 2017-07-22

**Authors:** Weijia Wang, Yan Zhang, Yong Yuan, Runqiang Yuan, Youye Yang, Xiuming Zhang, Dongmei Wen, Fuda Huang, Jinshu Wang

**Affiliations:** ^1^ Department of Laboratory Diagnosis, Sun Yat-Sen University Affiliated Zhongshan Hospital, Sun Yat-Sen University, Zhongshan 528403, PR China; ^2^ Key Laboratory of Diagnostic Medicine Designated by The Chinese Ministry of Education, Chongqing Medical University, Chongqing 400016, PR China

**Keywords:** NSC67657, ICAT, Wnt/β-catenin signaling pathway, HL60 cells, monocytic differentiation

## Abstract

NSC67657 is a new steroid drug that induces monocytic differentiation of acute myeloid leukemia cells. Here, we demonstrate that NSC67657 has opposing effects on expression of downstream targets of inhibitor of β-catenin and TCF (ICAT) and Wnt signaling in HL60 cells. ICAT binds to β-catenin, and this interaction is further increased in NSC67657-differentiated cells. ICAT overexpression decreases expression of Wnt downstream targets and increases sensitivity of HL60 cells to NSC67657, while ICAT silencing increases Wnt signaling and delays the NSC67657-induced cell differentiation. In addition, pharmacological inhibition of Wnt/β-catenin signaling increases the NSC67657-induced cell differentiation, while activation of Wnt/β-catenin signaling inhibits the differentiation, indicating Wnt/β-catenin signaling inhibits NSC67657-induced monocytic differentiation of HL60 cells. Our data demonstrate the opposing roles of ICAT and Wnt signaling in the NSC67657-induced monocytic differentiation, and suggest that ICAT and Wnt signaling may serve as therapeutic targets for leukemia chemotherapy.

## INTRODUCTION

Therapeutic strategies inducing cell differentiation offer conceptually elegant approaches to eradicate neoplastic cells from the human body. Successful induction of cell differentiation was first achieved with the introduction of retinoids for treating acute promyelocytic leukemia [1–3]. Harris et al. reported that sterol mesylate (NSC67657) was a potent activator of CCAAT enhancer binding protein alpha (C/EBPα) and could induce differentiation of HL60 cells into mature monocytes instead of granulocytes [4]; however, the responsible signaling pathways remain unclear.

All-trans retinoic acid and NSC67657 have been used to induce differentiation of HL60 cells to granulocytes and monocytes, respectively. Comparative proteomics studies identified the differentiation-specific protein ICAT and demonstrated its increased expression in differentiated HL60 monocytic cells [5]. ICAT is an 81 amino acid protein previously identified in a yeast two-hybrid screen, using the armadillo repeat region of β-catenin as a bait. ICAT inhibits β-catenin binding to T cell factor-4 (TCF-4), disrupts β-catenin/Tcf/DNA complexes, and decreases reporter gene activation by the β-catenin/ TCF-4 complex [6, 7].

β-catenin, a central protein in the Wnt pathway [8], plays essential roles in cell–cell adhesion and nuclear gene expression [9]. The canonical Wnt signaling is critical throughout vertebrate development, since it activates target genes that determine cell fate [8, 10]. Excessive β-catenin signaling has been implicated in various human cancers [11]. Gene activation is ultimately controlled by a transcriptional complex containing the DNA binding factor T cell factor (TCF) and β-catenin. In this complex, the COOH-terminal region of β-catenin serves as a transcriptional coactivator by recruiting components of the general transcriptional machinery, including TATA binding protein, proteins involved in chromatin modification, such as the histone acetyltransferase p300/cAMP binding protein, and a component of the SWI/SNF chromatin remodeling machinery, BRG-1 [6, 7, 12].

In hematopoiesis, β-catenin is involved in stem cell self-renewal and differentiation [13, 14]. It is expressed in primary human CD34+ progenitor cells and downregulated during myeloid differentiation, so that CD33+CD34- cells at the myeloblast stage have undetectable β-catenin levels. Primary acute myeloid leukemia (AML) blasts have a range of expression levels of β-catenin, apparently not correlating with CD34 expression, indicating that the link between β-catenin downregulation and myeloid differentiation is uncoupled in leukemia. In addition, β-catenin is detectable in the nuclear fraction of AML blasts, suggesting its involvement in transcriptional activation [15]. It is unclear whether ICAT and β-catenin are involved in differentiation of acute myeloid leukemia cells.

Previous studies have indicated that ICAT inhibition affects the β-catenin/TCF4 interaction and the downstream target genes in other tumor types. However, the ICAT/β-catenin signaling in acute myeloid leukemia cells remains incompletely understood. In addition, it remains unknown whether the Wnt/β-catenin signaling participates in the NSC67657-induced monocytic differentiation of acute promyelocytic leukemia cells.

## RESULTS

### NSC67657 induces monocytic differentiation of HL60 cells

**Figure 1:**
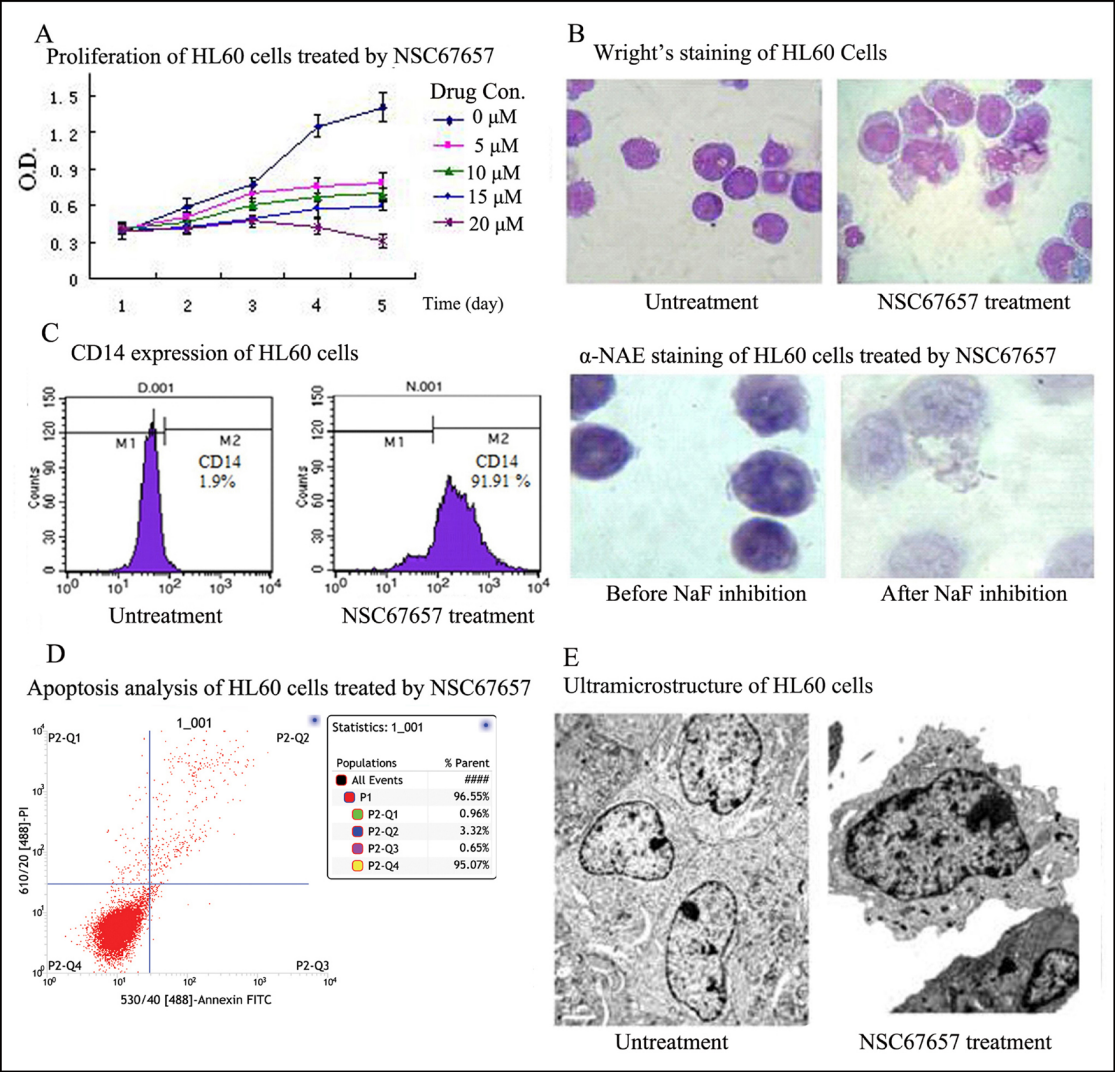
NSC67657 induces monocytic differentiation of HL60 cells **(A)** HL60 cells (1×10^5^ cells/ml) were incubated at 37°C for 5 d, with or without various concentrations of NSC67657. Proliferation of HL60 cells was assessed by the MTT assay. Judging from growth and differentiation states of the cells, 10 μM NSC67657 was the optimal drug concentration. **(B)** After 5 d treatment with NSC67657, HL-60 cells exhibited monocytic differentiation, with irregular nuclei and hypochromatic endochylema (arrow) (Wright’s stain, 400**×**). By esterase staining, the percent positive staining in the NSC67657 treated group was as high as 81.75±9.22%. This was decreased to 11.7% (inhibition rate > 50%) with NaF treatment (NAE stain, 400×). **(C)** HL60 cells treated with various differentiation inducers showed a time dependent increase in the proportion of differentiated cells. They were regarded as being completely differentiated after continuous treatment with selected drug concentrations for 5 d. At this time, the proportion of CD14 positive cells in the NSC67657 treated group was greater than 90%. **(D)** Apoptosis was evaluated by flow cytometry using fluorescein isothiocyanate-labeled annexin V. We found that few cells underwent apoptosis during HL60 cell differentiation. **(E)** Transmission electron micrographs (13,000 ×) of HL60 cells treated with NSC67657 (10 μM) or untreated. In untreated cells, chromatin was porous with the development of mitochondria. However, in drug treated cells, the heterochromatin was side-concentrated and azurophil granules were widely dispersed throughout the endochylema. Evidence of apoptosis, such as karyopycnosis, apoptotic bodies and vacuoles were not observed in any of the electron microscopic fields examined.

Proliferation of HL60 cells was significantly inhibited by treatment with 10 μM NSC67657 for 5 days (Figure 1A). The cell size increased, but there was no change in cell division and nuclei were irregular, without mutations. Alpha naphthol acetate esterase (α-NAE) activity was significantly inhibited by fluoride (>50%) and the percentage of CD14^+^ HL60 cells exceeded 90% (Figures 1B and 1C). These findings indicated that HL60 cells differentiated into monocytes. Furthermore, analysis of surface phosphatidylserine and ultramicrostructural observation showed no signs of apoptosis during cell differentiation (Figures 1D and 1E).

### NSC67657 increases ICAT expression, but inhibits Wnt signaling protein levels

**Figure 2:**
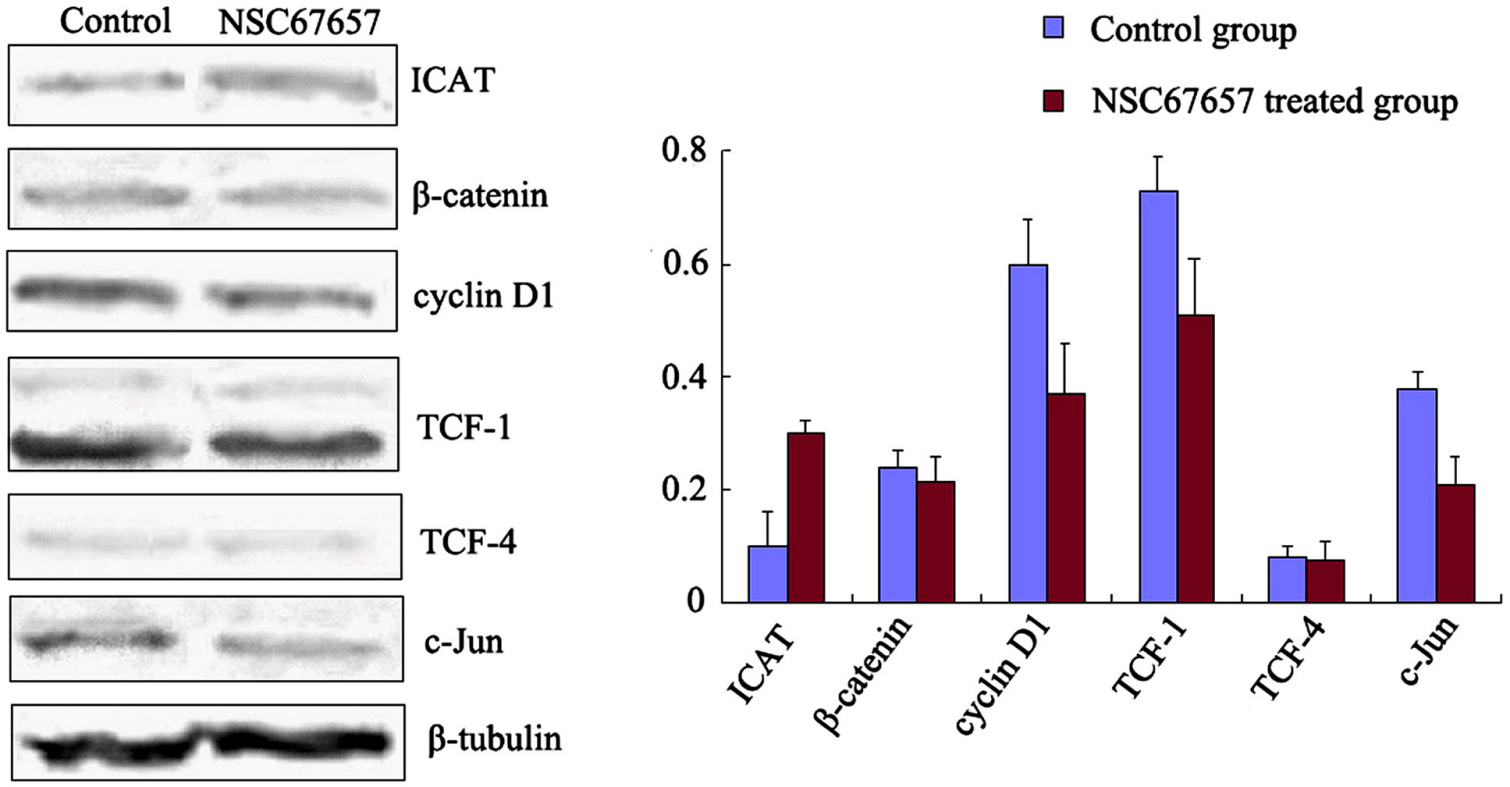
Expression of ICAT and Wnt/β-catenin signaling proteins during NSC67657-induced monocytic differentiation HL60 cells were treated with 10 μm NSC67657 for 5 d, and total protein was extracted and subjected to western blotting. In control samples, normal culture medium was added without drug, and total protein was extracted using the same procedures. Expression of ICAT protein in the NSC67657 treated group (0.31±0.02) was significantly higher than in the control group (0.09±0.06) (*P*=0.002). There was no difference (*P*=0.14) between β-catenin protein expression in the treated (0.22±0.03) and control (0.23±0.02) groups. Expression of cyclin D1 protein in the treated group (0.38±0.07) was significantly lower than in the control group (0.60±0.06) (*P*=0.001). Protein expression of TCF-1 in the treated group (0.58±0.07) was significantly lower than in the control group (0.74±0.05) (*P*=0.008). There was no difference (*P*=0.19) in TCF-4 protein expression in the treated (0.07±0.03) and control (0.08±0.02) groups. Protein expression of c-Jun in the treated group (0.21±0.05) was significantly lower than in the control group (0.41±0.03) (*P*=0.009).

ICAT protein levels in the NSC67657-treated group were increased compared to the control group (*P*=0.002). In contrast, expression of target proteins of the Wnt signaling pathway, cyclin D1, TCF1, and c-Jun, was decreased compared to the control group (*P*=0.01, *P*=0.03, and *P*=0.01, respectively). However, NSC67657 did not affect expression of β-catenin and TCF-4 proteins (*P*=0.14 and *P*=0.19), as shown in Figure 2.

### NSC67657 increases nuclear ICAT levels, while decreasing β-catenin, and inducing its cytoplasmic aggregation

**Figure 3:**
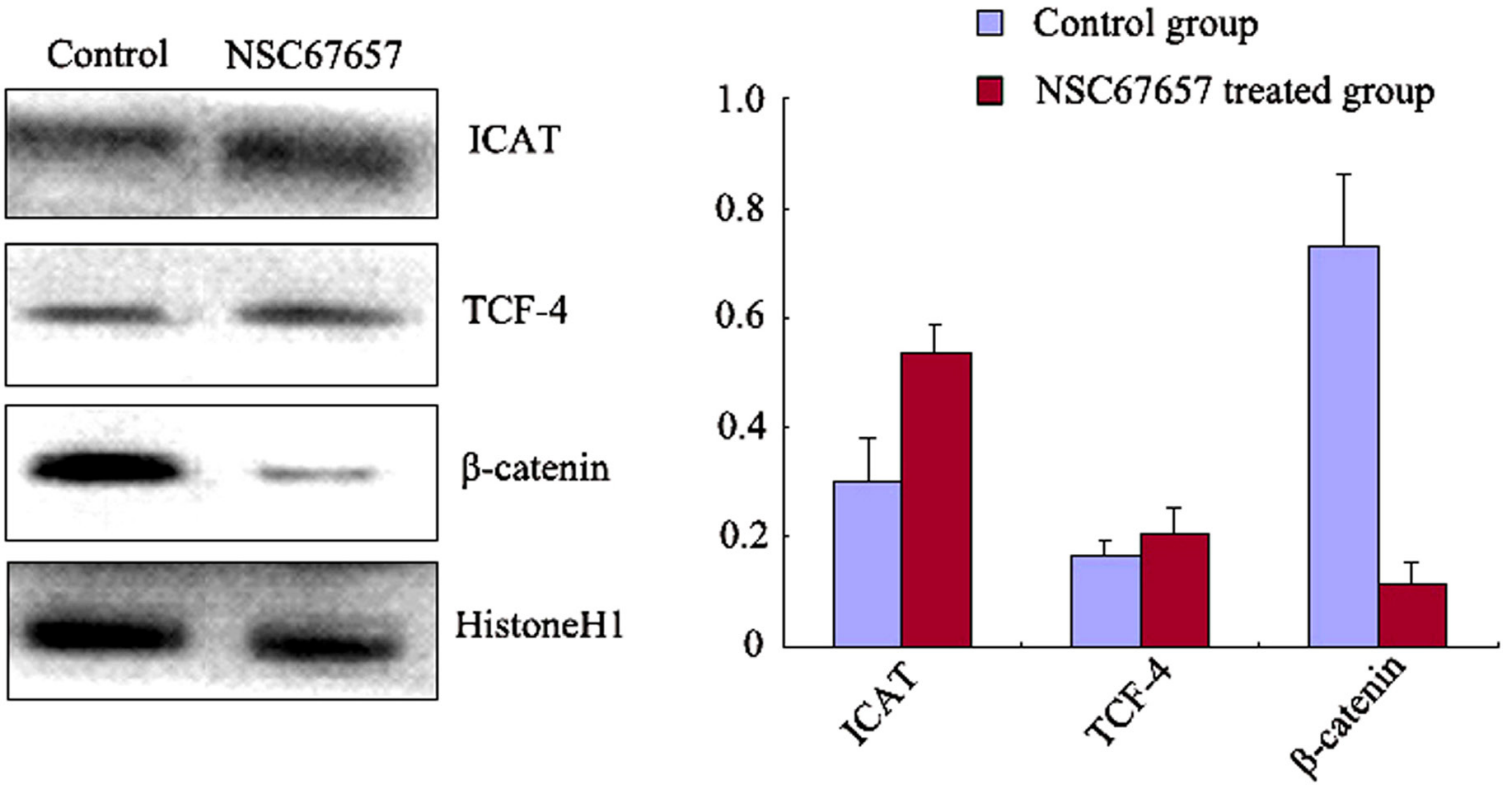
Nuclear protein levels of ICAT, β-catenin, and TCF-4 during NSC67657-induced monocytic differentiation HL60 cells were treated with 10 μM NSC67657 for 5 d and nuclear proteins extracted and subjected to western blotting. In control samples, normal culture medium was added without drug and nuclear proteins extracted using the same procedures. Nuclear levels of ICAT protein in the NSC67657 treated group (0.58±0.05) were significantly higher than in the control group (0.31±0.07) (*P*=0.001). There was no difference (*P*=0.16) in nuclear TCF-4 protein levels in the treated (0.24±0.04) and control (0.19±0.02) groups. Nuclear β-catenin protein levels in the treated group (0.13±0.04) were significantly lower than in the control group (0.68±0.12) (*P*=0.001).

**Figure 4:**
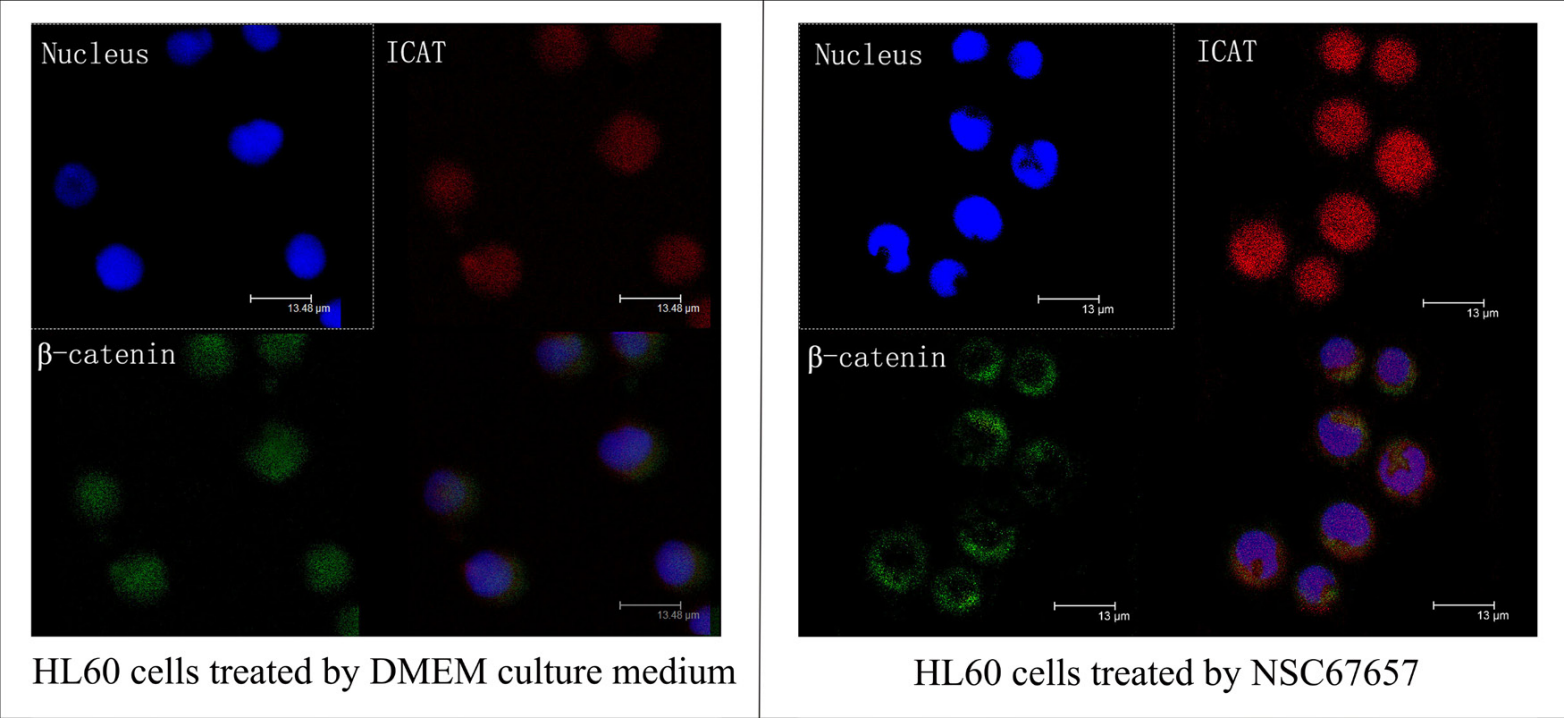
Immunofluorescence staining of ICAT and β-catenin during NSC67657-induced monocytic differentiation The upper left panel shows nuclei stained with DAPI (blue fluorescence). The upper right panel shows ICAT protein bound to TRITC-labeled secondary antibody (red fluorescence). The lower left panel shows β-catenin bound to FITC-labeled secondary antibody (green fluorescence). The lower right panel shows overlaid images for nucleus, ICAT and β-catenin staining in HL60 cells (8000×). The figure shows that, after differentiation of HL60 cells was induced by NSC67657, the intensity of ICAT protein staining in the nucleus and cytoplasm was increased, and that for β-catenin protein was almost undetectable in the nucleus and was aggregated in the cytoplasm.

NSC67657 increased the ICAT nuclear protein levels in HL60 cells (*P*=0.001), while it suppressed the protein levels of β-catenin (*P*=0.001); there was no change in the TCF-4 protein levels (*P*=0.12) (Figure 3). Immunofluorescence microscopy revealed an increased nuclear and cytoplasmic ICAT (red) staining in NSC67657-treated cells, while nuclear staining of β-catenin (green) was weak or absent. NSC67657 treatment also induced a green fluorescent staining of cytoplasmic aggregates (Figure 4).

### ICAT interacts with β-catenin in HL60 cells

**Figure 5:**
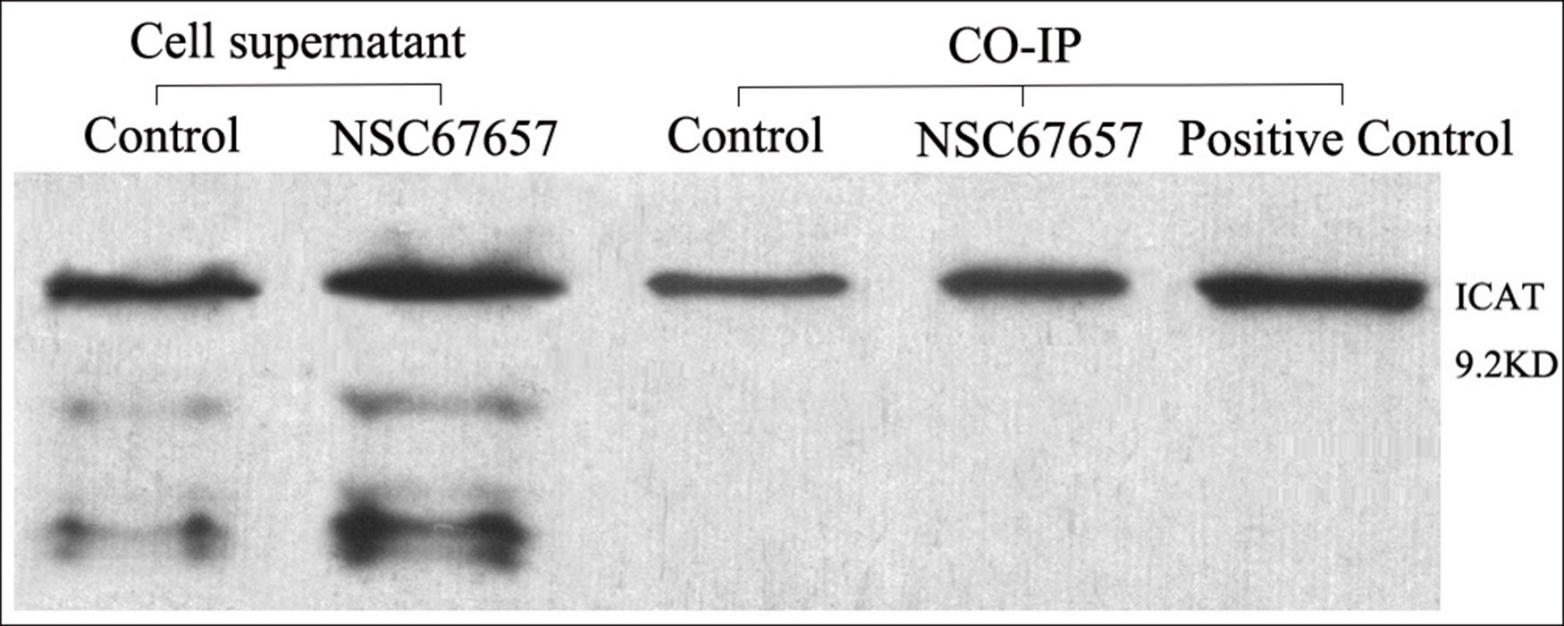
ICAT and β-catenin interaction analyzed by co-IP during NSC67657-induced monocytic differentiation The figure shows data from NSC67657 treated and control cells, collected in the logarithmic growth phase with cell concentrations adjusted to 1 × 10^6^ cells/ml. The prerequisite for success of this experiment was the presence of target protein in the supernatant of the cell lysates. With equivalent protein loading, the extent of protein–protein interaction could be estimated by densitometry. We found that the HL60 cells treated with NSC67657 had darker ICAT protein bands than the control group. This confirmed the presence of an interaction between β-catenin and ICAT proteins and that this interaction was increased with the drug treatment.

We used β-catenin-coated agarose beads for co-immunoprecipitation and detection of ICAT. ICAT was detected both in NSC67657-differentiated and undifferentiated HL60 cells. The ICAT levels in differentiated cells were higher than in untreated cells (*P*=0.01), indicating interaction between β-catenin and ICAT, and suggesting that the interaction between ICAT and β-catenin increases in differentiated cells (Figure 5).

### ICAT promotes NSC67657-induced monocytic differentiation of HL60 cells

**Figure 6:**
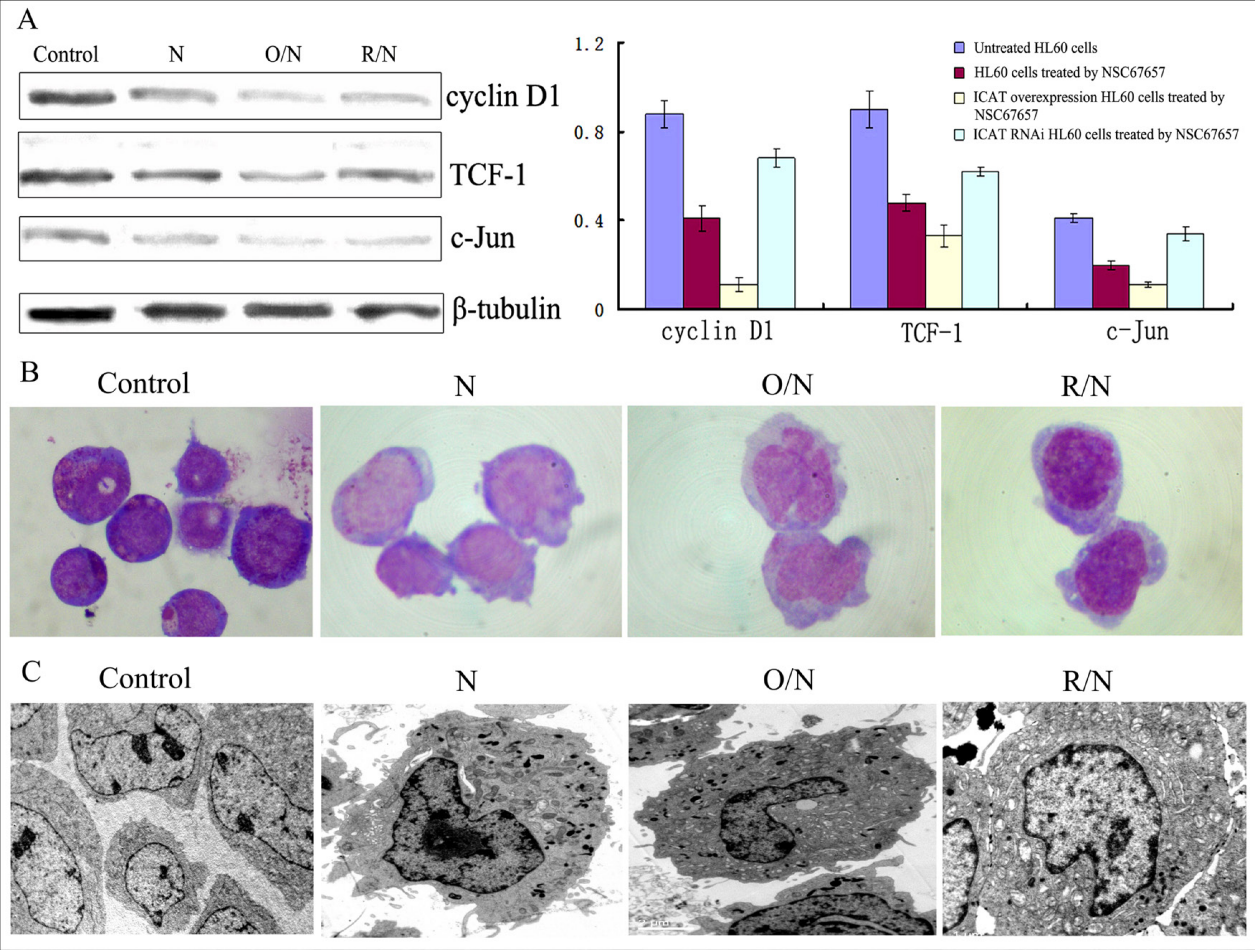
Effect of ICAT on NSC67657-induced monocytic differentiation The figure shows untreated HL60 cells (Control), NSC67657 treated cells (N), NSC67657 treated ICAT-overexpressing cells (O/N) and NSC67657 treated ICAT-silenced cells (R/N). **(A)** After 24 h drug treatment, levels of proteins related to the Wnt signaling pathway were lower in ICAT overexpressing models (*P*=0.02), but higher in ICAT-silenced models, compared to drug-treated normal cells (*P*=0.01). **(B)** Cellular morphology of drug treatment groups were observed using Wright's staining. The cytoplasm of drug treated normal HL60 cells was shallow, with protuberant pseudopodia and irregular edges and the nucleus was kidney-shaped. In ICAT-overexpressing HL60 cells, however, there were large cell bodies and more cytoplasm and a visibly irregular nuclear morphology, compared with in other treatment groups. In ICAT-silenced HL60 cells, the cytoplasm had a darker color and the nuclei were not significantly changed, but still immature. **(C)** Various groups of HL60 cells were treated with 10 μM NSC67657 for 24 h and ultrastructural observation (10,000×) conducted. Chromatin thickening and margination occurred in all drug-treated groups. Obvious azurophilic granules were present in ICAT-overexpressing cells, and there were also irregular nuclei, heterochromatic margination and swollen mitochondria. In addition, these cells showed significantly inhibited proliferation and a trend toward myeloid differentiation.

HL60 cells transfected with recombinant plasmid pDsRed-ICAT had increased ICAT protein expression (*P*=0.005) (data not shown), while HL60 cells transfected with RNAi-ICAT had decreased ICAT levels (suppression > 87%), validating our transfection protocol. NSC67657-treated cells overexpressing ICAT had decreased levels of Wnt signaling proteins, cyclin D1, TCF-1, and c-Jun (*P*=0.01). The percentage of CD14^+^ cells in cells overexpressing ICAT was 46.24±6.14%, significantly higher than in cells transfected with control plasmid (19.08±4.73%, *P*=0.003). In contrast, NSC67657-treated cells with suppressed ICAT expression had increased levels of the Wnt proteins (*P*=0.01). In these ICAT-silenced, NSC67657-treated cells, the percentage of CD14^+^ cells was 8.33±3.14%, significantly lower than in cells transfected with control plasmid (19.08±4.73%, *P*=0.0001). Cell morphology and ultrastructure were consistent with the CD14^+^ expression (Figure 6), and indicated that ICAT promotes the NSC67657-induced monocytic differentiation of HL60 cells.

### Wnt signaling inhibits NSC67657-induced monocytic differentiation of HL60 cells

Inhibition of Wnt/β-catenin signaling pathway by XAV-939 increased the proportion of CD14^+^ cells from 20.13±4.85% to 33.99±4.37%, in NSC67657-treated cells. In contrast, activation of Wnt/β-catenin signaling by LiCl decreased the number of CD14^+^ cells after NSC67657 treatment to 13.17±2.39%, indicating that the Wnt/β-catenin signaling inhibits the NSC67657-induced monocytic differentiation of HL60 cells.

## DISCUSSION

NSC67657 is a highly efficient inducer of monocytic differentiation [4]. The NSC67657- induced ICAT levels in HL60 cells suggested a link between the tumor suppressive effects of NSC67657 and deregulated Wnt signaling [5, 6]. Mutations involving components of the Wnt signaling cascade, especially in APC or β-catenin, are essential for initiation of many cancers, including colorectal cancer [16]. In the normal intestinal epithelium, these molecules are part of a multiprotein complex. In this complex, β-catenin is phosphorylated by GSK-3β and targeted for degradation by the ubiquitin-proteasomal pathway. Mutations in APC or β-catenin lead to dissociation of the complex, causing accumulation of unphosphorylated β-catenin, which translocates to the nucleus and acts as a transcriptional coactivator of TCF transcription factors [17, 18]. Activation of the Wnt/β-catenin/TCF signaling pathway promotes induction of downstream target genes, such as cyclin D1, c-*myc*, PPARδ, Tcf-1, matrilysin, and CD44 [19–23]. Induction of these genes dramatically affects cell and tissue development and oncogenesis [24, 25].

In our study, we found that the NSC67657-induced monocytic differentiation of HL60 cells increased ICAT protein levels and decreased Wnt/β-catenin signaling downstream targets. ICAT can inhibit the Wnt/β-catenin signaling pathway by binding to β-catenin and competing with its ability to bind the transcription factor TCF [6, 7]. Our study is the first to demonstrate the interaction between ICAT and β-catenin in acute promyelocytic leukemia HL60 cells, and show that this binding increases during monocytic differentiation. Interestingly, while the overall β-catenin levels were not significantly changed during NSC67657-induced differentiation, its nuclear levels decreased, and cytoplasmic aggregation appeared. This suggested that NSC67657 did not inhibit expression of the downstream target proteins of the Wnt/β-catenin signaling through β-catenin degradation [26]. In addition, these findings indicated that NSC67657 decreased the nuclear import of β-catenin, thus preventing its interaction with the TCF/LEF transcription factor family, and transcription of Wnt target genes [27]. However, β-catenin may be involved in monocytic differentiation, since myelomonocytic differentiation was impaired when mutant β-catenin was retrovirally transfected into normal progenitor cells [28]. Previous studies have demonstrated β-catenin nuclear translocation in mesenchymal ST2 cells, where JNK2 promotes nuclear translocation of β-catenin by phosphorylation at Ser-191 and Ser-605 [29, 30]. In APC mutant tumor cells, Pygo serves as a nuclear anchor protein that binds with BCL-1 and drives the nuclear entry of β-catenin [31, 32]. APC and axin can bind to β-catenin and transfer it to the cytoplasm via the nuclear membrane CRM1 receptor [33–36]. Cby, Lzts2, and other proteins are also involved in β-catenin cytoplasmic transport [37–39]. However, the mechanism of NSC67657-induced β-catenin cytoplasmic aggregation is unclear.

Our results indicate that ICAT inhibits the Wnt/β-catenin signaling during NSC67657-induced monocytic differentiation. ICAT may act as a bridge between NSC67657 and the Wnt/β-catenin pathway. Since modulation of the Wnt/β-catenin signaling affected the NSC67657-induced cell differentiation, these data indicate that the Wnt/β-catenin signaling is involved in the NSC67657-induced monocytic differentiation, even though additional mechanisms are likely to be involved. This is supported by our observation that, when either ICAT expression or the Wnt/β-catenin signaling was inhibited, HL60 cells were still partially differentiated, though their differentiation efficiency was greatly decreased. Future studies should determine whether ICAT is the only factor regulating the Wnt/β-catenin signaling during NSC67567-induced monocytic differentiation.

In conclusion, our study demonstrates that ICAT expression increases during NSC67657-induced monocytic differentiation, and indicates that while ICAT promotes the differentiation, Wnt signaling inhibits the NSC67657-induced monocytic differentiation. Together, our data suggest that ICAT and Wnt signaling may serve as therapeutic targets to modulate the differentiation of acute myeloid leukemia cells.

## MATERIALS AND METHODS

### Cell culture

Human promyelocytic leukemia HL60 cells were from the American Type Culture Collection (Manassas, VA, USA) and were maintained in Dulbecco's Modified Eagle's medium (DMEM) supplemented with 10% fetal calf serum (Invitrogen, Carlsbad, CA, USA), 2 mM glutamine and antibiotics (100 U/ml penicillin and 100 μg/ml streptomycin) at 37°C. The monocytic inducer NSC67657 was a kind gift from the National Cancer Institute (Bethesda, MD, USA). Cells (2.0×10^5^ cells/ml) were treated with 10 μM NSC67657. On day 3, cell cultures were split (1:2), NSC67657 was added to maintain its concentration, and cells were incubated for another 2 days.

### Cellular differentiation and apoptosis detection

3-(4, 5-dimethyl-thiazolyl-2)-2, 5-diphenyl tetrazolium bromide (MTT) assay was used to analyze cell proliferation, and cytochemical staining (Wright's and α-naphthl acetate esterase) was used to assess cell differentiation. The level of cellular differentiation was determined by detecting the cell surface CD14 antigen (Santa Cruz, CA, USA) with a fluorescence-activated cell-sorter (FACS) EPICSXL-MCL (Beckman/Coulter, Paris, France). Cell apoptosis was analyzed by flow cytometry using fluorescein isothiocyanate-labeled annexin V (TaKaRa, Kyoto, Japan). Ultra-microstructure was observed under a transmission electron microscope (Hitachi-7650, Tokyo, Japan).

### Western blotting

Whole cell extracts from HL60 cells were prepared, and protein concentration was quantified with the Bradford method. Samples containing 100 μg of protein were separated by 15% SDS-PAGE gel electrophoresis, and transferred to PVDF membrane. Membranes were blocked and incubated with primary antibodies, as indicated for each target protein, at 4°C overnight. Next, membranes were washed and incubated with horseradish peroxidase (HRP) labeled secondary antibodies, then developed with an ECL kit (Pierce, MA, USA), following the manufacturer's instructions. Quantity One 4.6.2 software was used for gray-value analysis of the electrophoresis bands in each group, to compare differential levels of ICAT, β-catenin and TCF-4 proteins and the Wnt signaling pathway downstream target proteins TCF-1, cyclin D1 and c-Jun. Rabbit anti-human β-catenin antibody was from Abcam (Cambridge, England), goat anti-human ICAT, α-tubulin, TCF4, cyclinD1, c-Jun and TCF1 antibodies were from Santa Cruz Biotechnology (Santa Cruz, CA, USA).

### Nuclear extract preparation and immunofluorescence microscopy

Nuclear proteins were extracted with the EpiQuik Nuclear Extraction Kit (Epigentek, CA, USA). Pre-cooled buffer, provided in the kit, was added in accordance with the packed cell volume, mixed and centrifuged. Pre-cooled nuclear protein extracting solution (1001 μl) was added to each pellet, representing the nuclear material and the samples were incubated in an ice bath for 30 min. After intermittent shaking, samples were centrifuged at 40 000g for 10 min at 4°C. The supernatant protein samples were then collected for further analysis. Using these methods, ICAT, β-catenin and TCF-4 proteins were analyzed.

HL60 cells, treated with NSC67657 for 5 d or untreated, were collected, fixed with cold acetone for 30 min then blocked with 150 μl/ml fetal bovine serum. After washed with PBS, samples were incubated with the appropriate primary antibodies overnight at 4°C. After washing again, appropriate RBITC or FITC labeled secondary antibodies and DAPI dye (Invitrogen, CA, USA) were added, with incubation times of 30 and 3 min, respectively, prior to observation under a confocal laser scanning microscope.

### Co-immunoprecipitation (co-IP)

HL60 cells, treated with NSC67657 for 5 d or untreated, were collected and cell lysis buffer and cell protease inhibitor Phenylmethanesulfonyl fluoride (PMSF) were added. After cells were lysed, 8 μg anti-β-catenin antibody was added and samples incubated, with slow shaking, overnight at 4°C. Protein A agarose beads (100 μl) were added to the samples with primary antibody in lysis buffer and tubes were incubated at 4°C, with slow shaking, for 2–4 h, enabling binding of the antibody and Protein A beads. Beads were then collected by centrifugation and washed at 4°C. Finally, 2× SDS sample loading buffer was added and samples boiled for 5 min, then centrifuged to obtain the supernatants. An anti-ICAT antibody was used to determine levels of ICAT by western blotting. Colon cancer cell associated SW480 protein was used as the positive control.

### Cell transfections

The pDsRed-ICAT recombinant plasmid was used, along with the following synthetic primers: sense 5'-GGGAATTCATGAACCGCGAGGGAGCAC-3' and antisense 5'-GGGGATCCCAGCTACTGGCCTCCGGTCTTCCGTCTC-3'. Lentiviral vector (GV248, hU6-MCS-ubiquitin-EGFP-IRES-puromycin) was provided by Shanghai Gene Chemistry Company. The interference target sequence was TCCGGAGGAGATGTACATT. The RNAi framework building synthetic sequence was sense 5'-ccggagTCCGGAGGAGATGTACATTctcgagAATGTACATCTCCTCCGGActtttttg-3' and antisense 5'-aattcaaaaaagTCCGGAGGAGATGTACATTctcgagAATGTACATCTCCTCCGGAct-3'. Recombinant plasmids (pDsRED-ICAT), which has been constructed before, was transferred into HL60 cells using electroporation technique. LV-ICAT-RNAi vector, which was firstly packaged and concentrated, then transfect HL60 cells. The transfection and interference efficiencies were identified. In HL60 cells, NSC67657 was used to produce ICAT overexpression and ICAT-RNAi to decrease ICAT expression. After 24 h of these treatments, expression of Wnt pathway downstream target proteins was analyzed and cell surface differentiation antigen CD14 expression, cell morphology and ultrastructure examined, before and after the drug treatments. This enabled assessment of the role of ICAT in NSC67657-induced cell differentiation.

### Modulation of Wnt signaling in NSC67657-treated HL60 cells

HL60 cells were harvested during the logarithmic growth phase. A portion of the cells was treated with10 μM NSC67657 for 24 h and then cells were harvested again by centrifugation. Another portion was treated with 20 μM XAV-939 (MCE, NJ, USA) for 3 d and cells were harvested again by centrifugation. In these cells, the supernatant was removed and 10 μM NSC67657 was added, followed by incubation for 24 h. A third portion of cells was treated with 20 mM LiCl for 24 h and cells collected by centrifugation. These were then treated with 10 μM NSC67657 for 24 h. For the control group, culture medium with no further additions was used, and cells were incubated for the same time periods under equivalent culture conditions. CD14 expression in all cells was detected with an upflow cell meter.

### Statistical analyses

Statistical analysis was performed using the program SAS9.4 (version 9.4; SAS Institute, Cary, NC). *P*-values < 0.05 were considered statistically significant. Differences in expression of cell surface differentiation antigens and other proteins, before and after HL60 cell treatments, were compared using a t-test.
